# Trends in missed presentations and late HIV diagnosis in a UK teaching hospital: a retrospective comparative cohort study

**DOI:** 10.1186/1471-2334-12-72

**Published:** 2012-03-28

**Authors:** Jared Wohlgemut, Timothy Lawes, Robert BS Laing

**Affiliations:** 1 School of Medicine and Dentistry, University of Aberdeen, Aberdeen, UK; 2 Infection Unit, Aberdeen Royal Infirmary, Aberdeen, UK

**Keywords:** HIV, Cohort studies, Delayed diagnosis, Adult

## Abstract

**Background:**

Late diagnosis is an important cause of HIV-related morbidity, mortality and healthcare costs in the UK and undiagnosed infection limits efforts to reduce transmission. National guidelines provide recommendations to increase HIV testing in all healthcare settings. We evaluated progress towards these recommendations by comparing missed opportunities for HIV testing and late diagnosis in two six year cohorts from North East Scotland.

**Methods:**

We reviewed diagnostic pathways of all patients newly diagnosed with HIV referred to infectious diseases and genito-urinary medicine services between 1995 and 2000 (n = 48) and 2004 to 2009 (n = 117). Missed presentations (failure to diagnose ≤ 1 month of a clinical or non-clinical indicator for testing), late diagnosis (CD4 < 350 cells/mm^3^), and time to diagnosis (months from first presentation to diagnosis) were compared between cohorts using *χ*^2 ^and log-rank tests. Determinants of missed presentation were explored by multivariate logistic regression. Breslow-Day tests assessed change in diagnostic performance by patient subgroup.

**Results:**

There were significant decreases in missed presentations (33% to 17%; *P *= 0.02) and time to diagnosis (mean 17 months to 4 months; *P *= 0.005) but not in late diagnosis (56% vs. 60%; *P *= 0.57) between earlier and later cohorts. In the later cohort patients were significantly more likely to have acquired HIV abroad and presented with early HIV disease, and testing was more likely to be indicated by transmission risk or contact with GUM services than by clinical presentation. Missed presentation remained significantly less likely in the later cohort (OR = 0.28, 95% CI 0.11 to 0.72; *P *= 0.008) after adjustment for age, transmission risks and number of clinical indicators. Reductions in missed presentation were greater in patients < 40 years, of non-UK origin, living in least deprived neighbourhoods and with early disease at presentation (*P *< 0.05). 27% of missed presentations occurred in primary care and 46% in general secondary care.

**Conclusions:**

While early diagnosis has improved in epidemiological risk groups, clinical indications for HIV testing continue to be missed, particularly in patients who are older, of UK origin and from more deprived communities. Increasing testing in non-specialist services is a priority.

## Background

Diagnosis is a critical limiting factor in the treatment and control of HIV/AIDS worldwide. Globally, up to 90% of people living with HIV may be unaware of their status [1]: with estimates ranging from 21% to 30% in developed nations [2,3]. In the context of a maturing pandemic, there has been a shift in attitudes towards testing, from an emphasis on protection of civil rights to improving public health, matched by models of provider, rather than patient, initiated testing [4]. Effectiveness of strategies for increasing testing and diagnosis is dependent upon regional epidemic status [1], but responses have been varied even within areas of low-HIV prevalence. Many countries, including the USA and Canada have recommended universal 'opt-out' testing in all general healthcare settings.

In the UK 25% of HIV infections are undiagnosed, undermining efforts to reduce transmission [5], and late diagnosis is an important cause of HIV-related morbidity [6], mortality [7] and healthcare costs [8]. A national audit of deaths among HIV infected adults found that late diagnosis was the leading remediable cause of HIV-related death [7]. The responsibilities of all health workers and services in reducing the diagnosis gap has been emphasised by leaders across disciplines [9]. In 2008 National guidelines for HIV testing were updated with the aim of increasing HIV testing in all healthcare settings [10]. Despite evidence of cost-effectiveness in low prevalence areas [11], routine 'opt-out' testing was not recommended, except in areas with adult prevalence > 0.2%. Instead, testing is to be offered universally in selected services, to those with epidemiologic risk-factors and to children or adults presenting with one or more prescribed clinical indicator diseases.

Beyond national surveillance [12], there is a need to describe progress towards national standards for testing and patterns in under-diagnosis at a regional level. Demographic changes in the local HIV population affected trends in missed acute HIV presentations in a study from Liverpool [13]. Increased testing has been associated with reductions in undiagnosed HIV infections in gay men in Scotland [14], but trends in diagnosis outside of high-risk groups are largely unknown.

This study compared the diagnostic pathways of HIV positive patients in North East Scotland diagnosed within two cohorts over a 15 year period, with the aim of describing trends in missed HIV presentations, late diagnosis and time-delay to diagnosis and associated risk-factors.

## Methods

### Study design and setting

Aberdeen Royal Infirmary (ARI) is a tertiary referral centre and acute teaching hospital, serving a population of 500,000 in the North East of Scotland (NHS Grampian). Outpatient HIV care is jointly provided by genito-urinary medicine (GUM) and infectious disease (ID) departments, with inpatient care in ARIs regional infectious diseases unit. Shared provision started in 1999, prior to which all patients were cared for by infectious disease specialists although HIV testing, including anonymous testing, took place in GUM throughout both time periods.

This comparative retrospective cohort study contrasted the diagnostic pathways of patients newly diagnosed with HIV and referred to GUM or ID departments at ARI between 1995 and 2000 (n = 48) with those diagnosed between 2004 and 2009 (n = 117).

### Definitions

Recommendations from the BHIVA guidelines (2008) were used to define clinical (presentations and haematological abnormalities) and non-clinical (service and epidemiological risk-groups) indicators for testing. First case-note or laboratory system record of any indicator was taken to be the first presentation. Declarations of past transmission risks or serological evidence of past infection, without prior documentation, were considered as presentations at time of recording.

The primary outcome was 'missed presentation', defined as failure to diagnose HIV within one month of a clinical or non-clinical indicator for testing. Secondary outcomes included, 'late' diagnosis (CD4 count < 350/mm^3 ^at diagnosis), 'very late' diagnosis (CD4 count < 200/mm^3 ^at diagnosis) and time to diagnosis (months from first presentation to diagnosis).

HIV clinical staging, and clinical diagnosis of AIDS, at first presentation and diagnosis were defined by World Health Organisation (WHO) criteria [15]. Patient postcode was linked to neighbourhood (small-area) Scottish Index of Multiple Deprivation (SIMD) decile and quintile [16] to provide a proxy for socio-economic status [17,18].

### Study population and data collection

All patients > 18 years of age, newly diagnosed with HIV and referred to infectious disease and GUM services in ARI within the two time periods were identified by anonymised-linked clinic records, patient management systems and notes of current or deceased patients. Patients moving outside of region were excluded as detailed clinical records were not available. Routinely collected data in case-notes from secondary care and electronic laboratory records were reviewed for each patient from earliest documented clinical or non-clinical indication for HIV testing (first presentation) to actual diagnosis. All presentations were documented up to point of diagnosis. Presentations to primary care were considered only where accompanied by documentation in electronic summaries from the patient's general practitioner (GP). Such summaries are typically provided on referral to, or on request from, secondary care in the region.

Research carried out was in compliance with section 25 of the Helsinki Declaration [19]. Permission to access medical notes was granted by the Clinical Effectiveness Facilitator from Aberdeen Royal Infirmary's Medical Records Department (Project ID: 2035).

### Data analysis

Frequency of missed presentation, late and very late diagnosis and time-delay to diagnosis were compared between the six-year cohorts using Fisher's exact and Log-rank tests. Cohort characteristics and indicators for testing at first presentation were compared using Fisher's exact or Mann-Whitney U tests. Risk factors for missed presentation were explored via univariate and multivariate logistic regression. Cohort (time period) was included as an *a priori *determinant and other covariates were entered into a multivariate model if *P *< 0.10 in univariate analysis. Breslow-Day tests were used to investigate heterogeneity in rates of missed presentation in later versus earlier cohorts across patient subgroups. Further comparison of time to diagnosis between cohorts was made using Kaplan-Meier curves and multivariate Cox-regression adjusting for case-mix.

Finally, in patients with a missed presentation we assessed disease progression by time of diagnosis by comparing the number of clinical indicators for testing, clinical stage and presence or absence of AIDS defining illness at first presentation and diagnosis using related-samples Wilcoxon-signed rank tests. Likelihood of immunologically advanced disease (CD4 < 200 cells/mm^3^) at diagnosis was compared between patients with and without missed presentation by logistic regression adjusting for baseline characteristics.

## Results

### Cohort characteristics

Compared with the earlier cohort, patients diagnosed between 2004 and 2009 were significantly more likely to have acquired HIV from outside the UK (59% vs. 37%; *P *= 0.01), be from less-deprived neighbourhoods and present with seroconversion illness or early HIV disease, despite comparable CD4 counts at diagnosis (Table 1).

**Table 1:** Demographic and clinical characteristics by cohort

	All n = 165	1995-2000 n = 48	2004-2009 n = 117	*P *value
Gender (female)	47 (29%)	10 (21%)	37 (32%)	0.163
Age, years	38 (10)	38 (9)	38 (11)	0.810
SIMD^†^, quntile				0.040
1 (most deprived)	24 (15%)	10 (21%)	14 (13%)	
2	22 (14%)	8 (17%)	13 (13%)	
3	38 (24%)	13 (27%)	25 (22%)	
4	38 (24%)	9 (19%)	29 (26%)	
5 (least deprived)	38 (24%)	8 (17%)	30 (27%)	
Origin				0.226
UK	108 (66%)	36 (75%)	72 (62%)	
sub-Saharan Africa	39 (24%)	9 (19%)	30 (26%)	
Other	18 (11%)	3 (6%)	15 (13%)	
Area of presumed acquisition				0.026
UK	78 (47%)	30 (63%)	48 (41%)	
Europe	10 (6%)	2 (4%)	8 (7%)	
sub-Saharan Africa	58 (35%)	14 (29%)	44 (38%)	
Asia/SE Asia	19 (12%)	2 (4%)	17 (15%)	
UK origin acquired in high-risk area.	26 (16%)	6 (12%)	20 (17%)	
Previous (negative) HIV test	51 (31%)	13 (27%)	38 (33%)	0.469
Months since last HIV test (if occurred)	41 (42)	29 (20)	45 (47)	0.090
Clinical staging at first presentation (WHO)				0.010
Primary HIV infection	3 (2%)	0 (0%)	3 (3%)	
Stage 1	102 (62%)	23 (48%)	79 (68%)	
Stage 2	11 (7%)	5 (10%)	6 (5%)	
Stage 3	24 (15%)	9 (18%)	15 (13%)	
Stage 4	25 (15%)	11 (23%)	14 (12%)	
Clinical staging at diagnosis (WHO)				0.002
Primary infection	2 (1%)	0 (0%)	2 (2%)	
Stage 1	84 (51%)	14 (30%)	70 (60%)	
Stage 2	12 (7%)	6 (13%)	6 (5%)	
Stage 3	29 (18%)	14 (29%)	15 (13%)	
Stage 4	38 (23%)	14 (29%)	24 (21%)	
Cd4 count at diagnosis (cells/mm^3^)				0.517
< 200	66 (40%)	21 (44%)	45 (39%)	
200-350	28 (17%)	8 (17%)	20 (17%)	
> 350	71 (43%)	19 (40%)	52 (44%)	

* *χ*^2 ^test of difference in proportion missed, *P *= 0.02. Otherwise non-significant. ^† ^Scottish Index of Multiple Deprivation.

### Primary outcomes

Overall, 22% of diagnoses were preceded by at least one missed presentation (n = 36). Frequency of late and very late diagnosis were 57% and 40% respectively and mean time to diagnosis was 8 months (95% CI: 4 to 12 months). There were significant decreases in missed presentations (33% to 17%; *P *= 0.02) and time to diagnosis (mean 17 months to 4 months; *P *= 0.005) between earlier and later cohorts, but not in frequencies of late (56% vs. 61%; *P *= 0.57) or very late (39% vs. 44%; *P *= 0.53) diagnosis.

### Indicators for testing at first presentation

**Figure 1:**
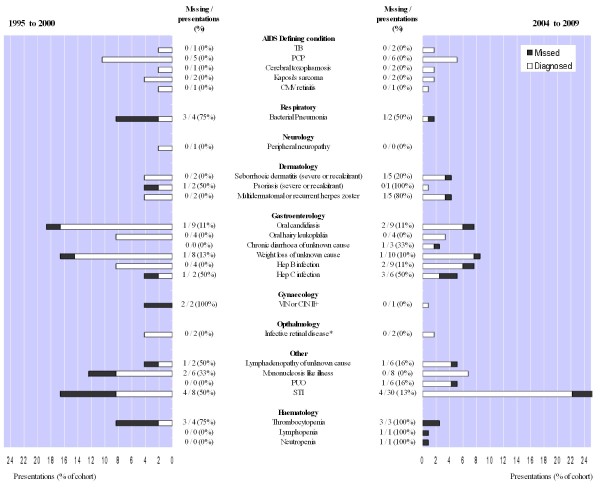
**Missed and diagnosed clinical presentations as percentage of total cohort**.

Patients in the later cohort were less likely to present with a clinical indicator disease (63% vs. 88%; *P *= 0.005) and more likely to present with epidemiological risk factors or via a service applying universal testing (Table 2). The higher proportion of patients acquiring HIV in high-risk regions (HIV prevalence > 1%) and increased contact with GUM services largely explained these trends. Significantly fewer first presentations with candidiasis (19% to 8%; *P *= 0.04) and bacterial pneumonia (8% to 2%; *P *= 0.04) were observed in the later cohort, otherwise clinical presentations were broadly comparable between the two cohorts (*P *> 0.10 for all) (Figure 1).

**Table 2:** Summary of indicators for HIV Testing at first presentation

	All n = 165	1995-2000 n = 48	2004-2009 n = 117	*P *value
**Summary**				
Median no. clinical indicators	1 (0-2)	1 (1-2)	1 (0-1)	0.002
Median number of all non-clinical indicators	2 (1-2)	1 (1-2)	2 (1-3)	< 0.001
Median number of all clinical & non-clinical indicators	3 (2-4)	3 (2-4)	3 (2-4)	0.697
**Services indicator (any)**	**102 (62%)**	**19 (40%)**	**83 (71%)**	**< 0.001**
Ante-natal/ToP	14 (9%)	4 (8%)	10 (9%)	0.964
GUM	61 (37%)	8 (17%)	53 (45%)	0.001
TB, Hep B/C, lymphoma	27 (16%)	8 (17%)	19 (16%)	0.964
Occupational health or screening*	11 (7%)	3 (6%)	8 (7%)	0.891
**Transmission risk indicator (any)**	**132 (80%)**	**33 (69%)**	**99 (85%)**	**0.021**
Country of origin high prevalence (> 1%)	53 (32%)	10 (21%)	43 (37%)	0.047
Men with disclosed sexual contact with men (MSM)	46 (28%)	14 (29%)	32 (27%)	0.813
IVDU	8 (5%)	2 (4%)	6 (5%)	0.794
Sexual contact abroad	42 (26%)	10 (21%)	32 (28%)	0.383
Partner HIV+	24 (15%)	6 (13%)	18 (15%)	0.633
Blood donors, dialysis patients or organ donors/recipients	6 (4%)	4 (8%)	2 (2%)	0.039
**Non-clinical indicator (any transmission risk or service indicator)**	**147 (89%)**	**37 (77%)**	**110 (94%)**	**0.002**
**Clinical Indicator diseases (any)**	**119 (72%)**	**42 (88%)**	**74 (63%)**	**0.005**

Indicators as recommended by national guidelines for HIV testing, except *.

### Missed presentations

Stratifying by clinical indicator disease (Figure 1), no presentations with AIDS defining illness were missed in either time period, whilst 8 of 9 blood dyscrasias were missed. *χ*^2 ^tests revealed a lower risk of missed presentation with sexually transmitted infection (STI) in the later cohort (13% vs. 50%; P = 0.02) and a non-significant decline in missed seroconversion illness (0% vs. 33%; P = 0.17). Presentations with pneumonia and hepatitis B or C were persistently missed. In patients not diagnosed at first presentation 13 (36%) had at least one further documented missed presentation before diagnosis. The majority of missed presentations occurred in non-specialist hospital services (47%) or primary care (27%), with 13% in GUM and 7% in antenatal services.

### Determinants of missed presentation

In univariate logistic regressions lower risks of missed presentations were predicted by age < 40 yrs, origin in high-risk regions, MSM, partner HIV positive, diagnosis in the later cohort and higher number of indicators for testing (Table 3). In a final multivariate model missed presentation remained significantly less likely in the later cohort (OR = 0.28, 95% CI 0.11 to 0.72; *P *= 0.008) after adjustment for these covariates. IV drug use was associated with a large, but non-significant increased risk of missed presentation.

**Table 3:** Risk-factors for missed presentation by univariate and multivariate logistic regression

	Univariate Regression		Multivariate Regression	
	
	OR (95% CI)	*P *value	OR (95% CI)	*P *value
Time period (later)	0.41 (0.19 to 0.89)	0.024	0.28 (0.11 to 0.72)	0.008
Age, per 10 years	1.79 (1.20 to 2.55)	0.004	1.69 (1.09 to 2.64)	0.007
Transmission risk:				
Country of origin high prevalence	0.35 (0.14 to 0.90)	0.029	0.39 (0.12 to 1.21)	0.103
MSM	0.45 (0.17 to 1.15)	0.097	0.30 (0.10 to 0.88)	0.028
IVDU	3.91 (0.92 to 16.5)	0.064	4.32 (0.82 to 23.0)	0.086
Partner HIV+	0.13 (0.02 to 1.01)	0.051	0.09 (0.01 to 0.81)	0.032
Any Service indicator	0.54 (0.25 to 1.03)	0.100	-^†^	
Number of clinical indicators	0.86 (0.62 to 1.20)	0.376	0.60 (0.37 to 0.97)	0.032
Number of non-clinical indicators	0.49 (0.32 to 0.75)	0.001	-*	
Number of all indicators	0.63 (0.46 to 0.87)	0.006	-*	

* Excluded as co-linearity with individual non-clinical or clinical indicators ^† ^Inclusion did not improve model fit.

**Figure 2:**
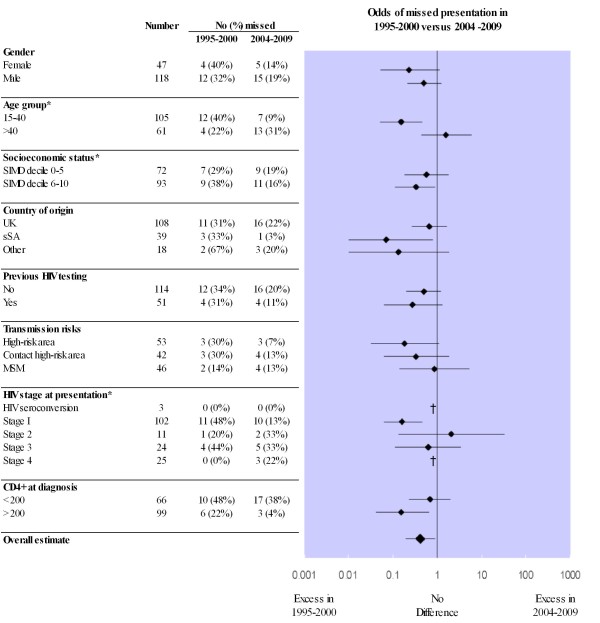
**Risk of missed presentation by time period stratified by clinical and epidemiological risk factors**. * Breslow-Day test of homogeneity of variance: *P *< 0.05 suggesting significant heterogeneity within the strata. ^† ^Not calculable due to small numbers. Transmission risks of IVDU, partner HIV positive, recipients of blood products/organs or dialysis patients were not calculated due to small numbers.

Stratifying by patient sub-group revealed a significantly greater reduction in risk of missed presentation in the later cohort amongst younger patients (< 40 yrs), living in least-deprived neighbourhoods and presenting with early or asymptomatic disease (Breslow-day tests; *P *< 0.05) (Figure 2). Improved diagnosis was also noted in those with a CD4 count > 200 at diagnosis, or from high-risk areas, although differences within strata were non-significant.

### Time to diagnosis

**Figure 3:**
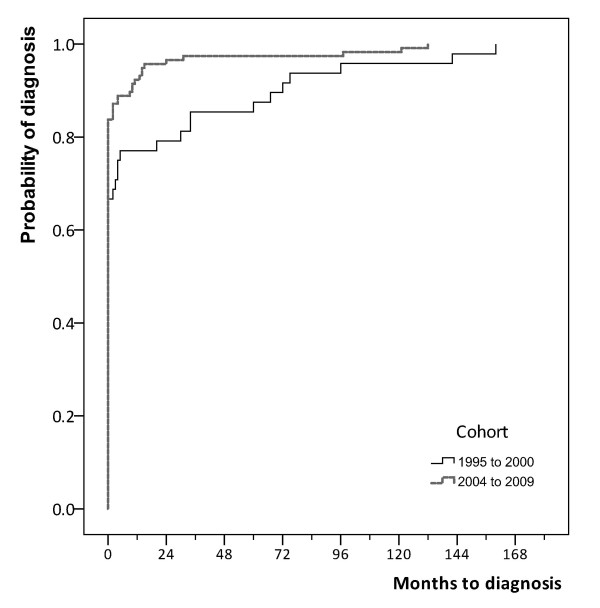
**Kaplan-Meier curves of time to diagnosis from first presentation by cohort**. (Log-rank test, *P *= 0.005).

Kaplan-Meier curves reflected a higher proportion of diagnoses made at first presentation in the later cohort as well as reduced time to diagnosis in those missed at first presentation (median delay 10 vs. 34 months; *p *= 0.005) (Figure 3). A multivariate Cox-regression model revealed a non-significant improvement in rate of (time to) diagnosis (adjusted HR = 1.36, 95% CI: 0.96 to 1.94; *P *= 0.084) after adjusting for case-mix: the proportion of patients diagnosed within one year of first presentation was 77% in the earlier cohort and 92% in the later cohort.

### Associations between missed presentation and disease progression

In those patients with a missed presentation (n = 36), the median number of clinical indicator diseases increased significantly from 1 at first presentation to 3 at diagnosis (Related-samples Wilcoxon-Signed rank tests, *P *< 0.001). Progression to AIDS (25%) or higher WHO clinical staging (53%) was common between missed presentation and diagnosis. Compared with those diagnosed at first presentation, patients with ≥ 1 missed presentation were also substantially more likely to have immunologically advanced disease (CD4 < 200 cells/mm^3^) at diagnosis: 74% vs. 30% (OR = 6.9, 95% CI: 3.0 to 16.2; *P *< 0.001).

## Discussion

This study looked for evidence of improvements in early HIV diagnosis in response to national strategies to increase testing in all healthcare settings. Diagnostic pathways were compared in two cohorts of newly diagnosed HIV patients from a 15 year period in North East Scotland. We demonstrated a significant decrease in missed presentations and time to diagnosis between the two time periods. However, patient groups, clinical presentations, and services in which opportunities for early diagnosis continue to be missed were also identified. Delay to diagnosis and missed presentation were strongly associated with disease progression at time of diagnosis.

As reported elsewhere in the UK [13], changes in socio-demographics of the HIV population had important impacts upon first presentations. We noted increases in patients from, or acquiring HIV in, regions of high HIV prevalence, and living in less deprived neighbourhoods. In North East Scotland these trends are related since contact with hyperendemic regions is largely related to a mobile labour-force in the oil industry [20]. An important minority diagnosed in occupational health screening emphasises the importance of HIV care for workers in globalised industries [21]. Patient ethnicity is an important epidemiological risk factor that should be recorded at first contact with all UK healthcare services [10]. Substantial reductions in missed presentations in those acquiring HIV in high-risk regions, suggested increased awareness of transmission risks, while patient factors, including access to alternative health insurance, may explain discordance with findings of barriers to diagnosis for some migrant populations [22].

Our findings also warn against stereotyping the 'at risk' patient [23]: 20% of diagnoses occurred in those without transmission risks. Missed presentations persisted in patients who were older, of UK origin and from more deprived communities. Delayed HIV diagnosis in older patients is common [13,23] and of national importance. Across the UK the proportion of new diagnoses made in those > 40 years increased from 23% to 38% between 1995 and 2009[24] and older age predicts poorer outcomes from anti-retroviral therapy, particularly for immunologically advanced disease [25]. Overemphasis on ethnicity, without reference to contextual factors such as deprivation and social networks, impair effective prevention of other infectious diseases including sexually STIs [26] and tuberculosis [27]. Regional patterns in transmission risks may be of more importance to clinicians than national epidemiology. A high proportion of heterosexual and IVDU transmission amongst those of UK origin has been noted in the North East of Scotland [20]. Under-diagnosis in IVDUs may be particularly important given associations with high-risk sexual behaviours [28] and TB transmission [29].

In common with a study of acute HIV presentations from Liverpool [13], we found consistent patterns of all presentations in both cohorts. Despite this we found limited evidence that strategies to increase HIV diagnosis have improved our ability to recognise early clinical indicators in the absence of epidemiological risk-factors. In particular, unexplained haematological abnormalities, bacterial pneumonias and viral hepatitis frequently failed to prompt HIV testing. This may reflect the frequency of these presentations in non-specialist services and immunocompetent patients. HIV related thrombocytopenia is common and not isolated to disease stage or risk group but is rarely profound or symptomatic [30]. Hepatitis C acquisition may be highest around the time of primary HIV infection [31]. Automatic flags for HIV testing on laboratory reports may help clinicians identify these presentations. Non-specificity of early HIV presentations remains a clinical challenge: while all AIDS defining illnesses prompted immediate diagnosis, we identified several commonly missed clinical indicators including, recurrent herpes zoster [32] oral candidiasis [33] and weight loss. By contrast recognition of primary HIV infection was better than reported elsewhere in the UK [34].

In common with previous studies [13,35] missed presentations were noted in both specialist and non-specialist services. Universal screening in services specified by national guidelines would have captured only 62% of new diagnoses and frequency of multiple missed presentations suggest need for improved HIV awareness at all care levels. The 27% of missed presentations identified in primary care supports emphasis on improving diagnosis in this setting [22]. A marked increase in contact with GUM services explains a significant improvement in diagnosis following an STI. The GUM model of care with anonymised records, drop-in facility and routine HIV testing may offer significant advantages in early HIV diagnosis, particularly if planned integration with primary care is realised [36].

The association of missed presentation and delay to diagnosis with immunological and clinical disease progression is well documented [37]. It is encouraging to note that the median time delay from missed presentation to diagnosis had fallen by 24 months between the first and second cohort. The disparity between reductions in missed presentation and non-significant decline in late or very late diagnosis may reflect shifts in demographics of all patients. In particular under-diagnosis and lack of documented health contact outside the UK create challenges to detection of HIV in migrants. Debate around the cost-effectiveness and practicability of universal 'opt-out' testing continues in the UK [38]. However our findings suggest that opportunistic testing presents considerable challenges to clinicians and public health. When opportunities to prevent transmission are added to those of improved survival after early diagnosis, the argument for routine HIV surveillance is persuasive [11,38].

The generalisability of findings from this study may be limited by regional differences in HIV populations, testing and care provision arrangements. However, we believe the study highlights important challenges in opportunistic testing relevant to UK and similar healthcare settings. Limits to the internal validity of our findings arise from a retrospective design reliant upon records in secondary care. It was not possible to identify or include patients lost to follow up, or relocating out of area. A previous study of new HIV diagnoses between 1985 and 1997 suggest over representation of those of non-UK origin, students and prisoners in those discontinuing care within region [20]. Incomplete documentation in case-notes may mean failure to capture all relevant presentations or risk factors. However, systematic bias was minimised by a standardised data collection method for all patients with independent assessments of relevance of presentations by two investigators. Presentations in primary care were identified from printed summaries or documentations in case-notes from secondary care only, and may be under-represented. Investigation of missed presentations in this setting is an important priority [22]. Without concurrent control it is not possible to attribute trends in missed presentation to national strategies to improve HIV diagnosis [39]. Indeed our study suggests most improvements relate to changes in patient demographics and service provision. Further prospective, cluster randomised controlled trials of interventions to improve HIV diagnosis in general healthcare settings are required.

## Conclusions

Early diagnosis of HIV infection is a priority in HIV-related healthcare, but is complicated by changing socio-demographics of HIV populations in the UK. A significant reduction in missed presentations in North East Scotland was attributable to increased recognition of epidemiological risk-factors and contact with GUM services, rather than improvements in opportunistic testing of patients with clinical indicators. Consequently, missed opportunities for early diagnosis persist in populations considered at lower risk and presenting to non-specialist services. Diagnosis in those with haematological abnormalities or other blood-borne viruses might be improved by including advice on HIV testing in laboratory reports. However, evidence of disease progression after missed presentation further suggests the need to reconsider routine 'opt-out' testing in all general healthcare settings to reduce delays to HIV diagnosis.

## Competing interests

The authors declare that they have no competing interests.

## Authors' contributions

JW and RBSL conceptualised the study. JW, RBSL and TL were involved in the study design. JW and TL collected data. TL performed the statistical analysis. TL, RBSL and JW drafted the manuscript. All authors read and approved the final manuscript.

## Pre-publication history

The pre-publication history for this paper can be accessed here:

http://www.biomedcentral.com/1471-2334/12/72/prepub
